# Post-Ebola sequelae among Ebola child survivors in Sierra Leone

**DOI:** 10.1186/s12887-021-02957-w

**Published:** 2021-10-30

**Authors:** Claudette Amuzu, Peter Bai James, Abdulai Jawo Bah, Alex Vandy Saffa Bayoh, Shepherd Roee Singer

**Affiliations:** 1 Braun School of Public Health, Hadassah/Hebrew University, Jerusalem, Israel; 2 National Centre for Naturopathic Medicine, Faculty of Health, Southern Cross University, Lismore, Australia; 3 Faculty of Pharmaceutical Sciences, College of Medicine and Allied Health Sciences, University of Sierra Leone, Freetown, Sierra Leone; 4 Queen Margaret University Edinburgh, Musselburgh, Scotland, UK; 5 ICAP Columbia University, Freetown, Sierra Leone; 6 Israel Ministry of Health, Jerusalem, Israel

**Keywords:** Ebola, Ebola survivors, Pediatric, Long term sequelae, Sierra Leone

## Abstract

**Background:**

There are limited data regarding the long-term health effects of child survivors of the 2013-2016 West African Ebola virus disease (EVD) outbreak. Here, we assess post-Ebola sequelae among EVD child survivors by comparing the self-reported symptoms between EVD child survivors and their close household contacts over one year after the end of the outbreak.

**Methods:**

EVD child survivors(n=159) and their close contacts(n=303) were enrolled in Western and Eastern Sierra Leone. Demographics and self-reported symptoms data were collected using an interviewer-administered questionnaire. We compared a list of self-reported symptoms between EVD child survivors and their close household contacts using backward stepwise logistic regression.

**Results:**

EVD child survivors were more likely to be orphans compared to their close contacts. Musculoskeletal, ocular, auditory and neurological symptoms were more prevalent among Ebola child survivors than their close contacts (p<0.001). Joint pain and headache were the most common self-reported symptoms in EVD child survivors and their close contacts. Joint pain (AOR=2.633; 95 % CI:1.31-5.28, p=0.006), eye pain (AOR=4.56;95 %CI: 2.16-9.64, p<0.001), hearing loss (AOR=3.85; 95 %CI: 1.15-12.87, p=0.029), memory impairment (AOR=7.76;0.95 %CI: 1.34-45.01 p=0.022), mood changes (AOR=5.07; 95 %CI: 2.35-10.94, p<0.001) were more common among survivors than their contacts.

**Conclusions:**

Our data suggest that EVD child survivors have higher odds than their close contacts of suffering from musculoskeletal, ophthalmic, auditory and neurological impairment more than a year after the end of the EVD outbreak. Routine screening, treatment and monitoring of these symptoms is required to prevent long-term disability among EVD child survivors.

**Supplementary Information:**

The online version contains supplementary material available at 10.1186/s12887-021-02957-w.

## Background

The recent West African Ebola virus disease (EVD) epidemic began in 2013 in Guinea and spread rapidly to Liberia and Sierra Leone. These three areas were classified as countries with intense transmission by the World Health Organisation (WHO) due to their high infection rates [1]. The total number of cases and deaths from this epidemic is the highest ever recorded for Ebola, with 28,652 and 11,325 respectively, of which 28,616 cases and 11,310 deaths were reported from these countries [2]. The first confirmed case of EVD in Sierra Leone was documented in May 2014 in the Eastern District of Kailahun and subsequently spread to all thirteen Districts. The total number of EVD cases (confirmed, probable, and suspected) in Sierra Leone recorded at the end of the West African outbreak was 14,124 [2]. Of these, the total number of laboratory confirmed cases was 8,706, and the total number of deaths was 3,956 [2]. Due to its magnitude, the West African EVD outbreak recorded the highest number of survivors, and WHO estimated the figure to be approximately 10,000 [3]. In Sierra Leone, close to 4000 EVD patients are known to have survived the disease. Previous studies in Sierra Leone, Liberia, Guinea and the Democratic Republic of Congo have found physical and mental health sequelae to be common among EVD survivors. These sequelae include chronic joint and muscle pain, fatigue, anorexia, hearing loss, blurred vision, headache, sleep disturbances, psychiatric and mood disorders, short-term memory problems, and uveitis, among others [4–6]. For instance, a retrospective cohort study in Uganda reported that Ebola survivors compared to their close contacts were at higher risk of developing arthralgia, ocular symptoms (retro-orbital pain, blurred vision), hearing loss and difficulty sleeping [7]. Also, an observational cohort study in Liberia showed that headache, joint pain, memory loss, muscle pain and fatigue were more common among Ebola survivors than among controls [8]. In addition, a recent Sierra Leonean study conducted in Kenema indicated that musculoskeletal symptoms (joint pain, muscle pain, joint tenderness etc.), ophthalmologic, auditory, psychiatric, and constitutional symptoms were more prevalent among Ebola survivors than their contacts [9].

From the WHO’s Interim Guidance for clinical care for survivors of EVD, all the systems in the body are affected by the sequelae of Ebola [3]. The major challenge facing survivors is often the need for specialised services, which are not readily available in the affected countries [10, 11]. These include ophthalmic care and mental health services. While documentation of sequelae in adults has improved, it is becoming apparent that there is limited information on sequelae available for EVD child survivors. Among nations affected by the outbreak, Sierra Leone recorded the highest rate of children infected with Ebola, with 20 % of confirmed infections aged 14 years or younger [12]. Case fatality rate (CFR) in children younger than five years was high in the West African EVD epidemic, with children less than one year having a case fatality ratio of 90 % and children under five years having a CFR of 80 % [13].

There is limited information available on sequelae for EVD child survivors, although the physical, psychological, and social impacts of the disease on this vulnerable group are significant [4]. Currently, only three post-Ebola sequelae studies included children and adult EVD survivors as part of their target population [8, 9, 14]. Only one study compared post-Ebola sequelae between children and adult, and in this study, more adult than children were reported to show musculoskeletal, ocular and other clinical sequelae [14]. So far, there are no studies that have explored post-Ebola sequelae only among EVD child survivors. Also, there are no known age-specific interventions to prevent complications in EVD child survivors. The dearth of information on complications in EVD child survivors creates a gap in the literature on Ebola and limits the ability to offer appropriate follow-up and care to these children. Therefore, it is important to identify and evaluate EVD child survivors in Sierra Leone, create a cohort that can be followed up, and inform preparation for future outbreaks. This study assesses post-Ebola sequelae among EVD child survivors by comparing the self-reported symptoms between EVD child survivors and their close household contacts.

## Methods

### General Settings

Sierra Leone has an estimated population of 7 million, of whom approximately 70 % live below the poverty line [15]. The country’s indices for maternal and infant mortality are among the worst globally [16]. This is partly due to the period of civil conflict (1991–2002) that devastated the country and its health system. Even before the Ebola outbreak, there were only two doctors and 17 nurses per 100,000 population, most of whom were situated in urban areas [15]. The health infrastructure is tiered into tertiary hospitals, district hospitals, and peripheral health units (PHUs) designed to deliver primary health care. The PHUs include community health centres (CHCs), community health posts (CHPs) and maternal and child health posts (MCHPs) [17].

The study was conducted in the Western Area urban and rural districts in the Western region and Kenema district in the Eastern region of Sierra Leone. The Western Area is the wealthiest region in Sierra Leone, having the largest economy, financial and cultural centre, as well as the seat of the country’s national government. It is divided into two districts: Western Area Rural and Western Area Urban. Kenema district is the district headquarter of the Eastern Province of Sierra Leone. These two regions were regarded as epicentres, with up to 4000 cases of Ebola reported in total [2].

### Study design, population, sampling, and data collection

A cross-sectional study was conducted among EVD child survivors and their close contacts between January and April 2017. EVD male and female child survivors aged 5-17years were enrolled in this study. We excluded those less than five years and those who could not properly respond to some of the questions. These included those with high fever and those experiencing acute emotional distress. We excluded respondents aged less than five years and those showing signs of acute emotional distress because they could not meaningfully respond to the survey questions. Given that high fever is a cardinal sign of EVD, we excluded respondents presenting with such a sign as a precautionary measure to prevent risk of EVD infection for data collectors.

**Fig. 1:**
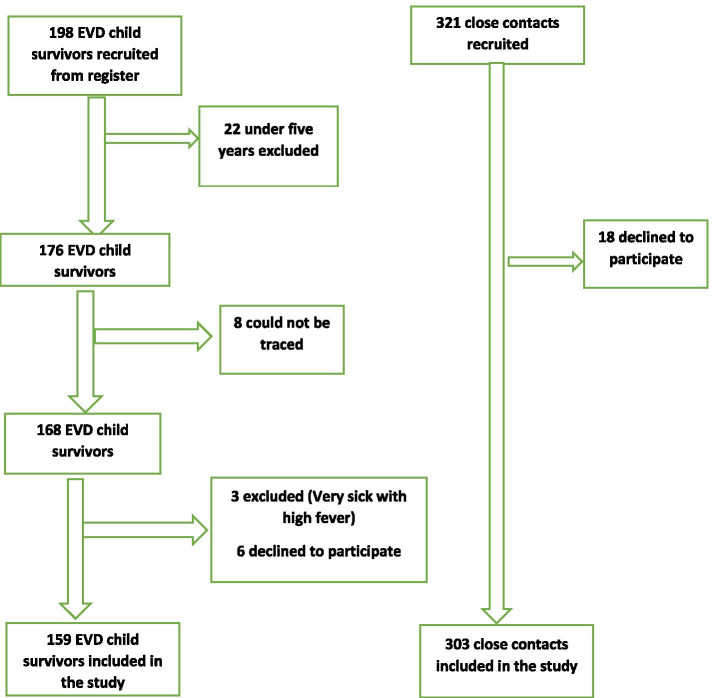
- Flow chart of EVD child survivors and their close contacts’

EVD child survivors were identified based on a list obtained from a registry of Ebola survivors from the Ministry of Social Welfare, Gender and Children’s Affairs (MSWGCA) and Sierra Leone Association of Ebola Survivors. Controls used were child contacts of EVD child- survivors from the same household who were not infected with EVD. They were matched with EVD child survivors by age and sex. These close contacts provided a healthy cohort with similar genetic, socioeconomic, and environmental characteristics to their EVD child survivor cohort. Two household contact were chosen for every EVD child survivor. A cohort of 198 EVD child survivors was obtained from the registry; 22 were excluded as they were under the age of five. We could not trace eight of them, three were excluded because they were sick with high fever, and six declined to participate. The study profile of EVD child survivors and their close contacts is shown in Fig. 1.

### Data variables and source

Trained personnel collected information from survivors, contacts or parents/guardians of survivors and controls using a pre-designed questionnaire (Additional file 1). The questionnaire was designed based on the available literature regarding post-Ebola sequelae among adult Ebola survivors [6, 18–20]. Data were collected through interviewer-administered format, and interviews were conducted in Krio (a widely spoken language in Sierra Leone) at participants’ place of residence. The following information was collected; age, sex (male vs. female), weight, geographical location (Western Area Urban, Western Area Rural and Kenema), social status (orphan vs. non-orphan), educational level (no formal education, primary and secondary), how many times they were hospitalised during and after the Ebola outbreak, history of any disease before EVD. We also collected data on post-Ebola symptoms of EVD based on the available literature [4, 6, 19–21].

### Statistical analysis

Data from filled questionnaires were coded and analysed using Statistical Package for Social Sciences (SPSS) for Windows, Version 23 (Chicago Inc.). Categorical and continuous variables were represented in frequency, percentages, mean, and standard deviation, respectively. Bivariate analysis using Chi-square or Fisher exact tests were used to establish an association between survivors and controls. A backward stepwise multivariate regression analysis was used to determine the odds of presenting with a particular post-Ebola symptom between child survivors and their contacts. Age, sex, gender, weight, education level, geographical location, and social status (orphan vs. non-orphan) were considered potential confounders and were controlled when conducting the regression analysis.

### Ethical approval

 Permission to conduct this study was obtained from the Sierra Leone Scientific and Ethics Review Committee, Ministry of Health and Sanitation, Freetown. Verbal and written informed parental/guardian consent were obtained for all participants using the WHO Research Ethics Committee (WHO ERC) template for research involving children.

## Results

One–hundred and fifty-nine EVD child survivor cases and 303 closed contacts finally participated in our study. The overall average age of respondents (EVD child survivors and close contacts) was 10.09±3.6. The minimum and maximum age for each cohort was five and 17, respectively. The average age of EVD child survivors was higher than their close contacts (11.08±3.68 vs9.56±3.38; p<0.001). Also, the mean weight of EVD child survivors was higher than their contact (38.5±16.4 vs. 32.6±14.2; p<0.001). Approximately half of all respondents were females (n=236, 51.1 %) with similar proportion among EVD child survivors (n=81, 50.9 %) and close contacts (n=154, 51.2 %). There was no statistically significant difference between survivors and controls with respect to gender and location of participants. More EVD child survivors were orphans compared to their close contacts (n=83,52.2 % vs. n=43,14.2 %; p<0.001). Likewise, more EVD child survivors had attained secondary education than their close contacts. Also, more EVD child survivors were hospitalised three or more times after the end of the EVD outbreak compared to their close contacts (n=105, 67.3 % vs. n=51, 17.0 %; p<0.001). Please see Table 1 for details.

**Table 1:** Socio-demographic profile of EVD child survivors and close contacts

Characteristics	Variables	EVD child Survivor n (%) N=159	Close contacts n (%) N=303	Total n (%)	p-value
Age(years)^a^	Mean ±SD (range)	11.08±3.68	9.56±3.38(5-17)		<0.001
Sex	Male	78(49.1)	148(48.8)	226(48.9)	0.958
	Female	81(50.9)	154(51.2)	236(51.1)	
Weight(kg)^a^	Mean	38.5±16.4	32.6±14.2		<0.001
Location of Participant	Western Area Urban	75(47.2)	143(47.2)	218(47.2)	0.983
	Western Area Rural	53(33.3)	99(32.7)	152(32.9)	
	Kenema	31(19.5)	61(20.1)	92(19.9)	
Social Status	Orphan	83(52.2)	43(14.2)	126(27.3)	<0.001
	Non-Orphan	76(47.8)	260(85.8)	336(72.7)	
Education Level	None	16(10.1)	46(15.2)	62(13.4)	<0.001
	Primary	93(58.5)	224(73.9)	317(68.6)	
	Secondary	50(31.4)	33(10.9)	83(18.0)	
History of illness prior to Ebola	Yes	47(29.6)	49(16.2)	96(20.8)	0.001
	No	112(70.4)	254(83.8)	366(79.2)	
How many times hospitalised before Ebola	Less than three times	139(88.5)	281(93.0)	420(91.5)	0.113
	Three or more times	18(11.5)	21(7.0)	39(8.5)	
How many times hospitalised during Ebola outbreak	Less than three times	140(88.1)	296(97.7)	436(94.4)	<0.001
	Three or more times	19(11.9)	7(2.3)	26(5.6)	
How many times hospitalised after the end of Ebola	Less than three times	51(32.7)	249(83.0)	300(65.8)	<0.001
	Three or more times	105(67.3)	51(17.0)	156(34.2)	

^a^Student t-test was used to determine the difference between means

Table 2 shows the clinical profile of EVD child survivors and their close contacts. Musculoskeletal, ocular, auditory and neurological symptoms were more prevalent among EVD child survivors compared to their close contacts. Close to two-thirds of EVD child survivors (n=101, 63.5 %) reported experiencing joint pain compared to less than one-fourth (n= 58, 19.1 %) among their close contacts. Also, muscle pain was reported in more than a third of EVD child survivors (n=57,35.8 %) compared to less than a fifth of their close contacts n=39,12.9 %). Close to one in five EVD child survivors (n=29,18.2 %) reported to experienced blurred vision compared to less than one in 30 of their close contacts (n=11,3.6 %). One in five EVD child survivors (n=33,20.8 %) presented with hearing loss compared to one in 30 close contacts (n=9,3.0 %). In addition, one in ten EVD child survivors (n=17, 10.7 %) compared to one in 100 close contacts (n=4,1.3 %) experienced tremors. Close to half of EVD child survivors(n=72,45.3 %) compared to one in 20 close contacts (n=18,5.9 %) experienced mood changes. Headache [n= 139(86.9)] followed by joint pain[n=101(63.1 %)] were the most common self-reported symptoms among EVD child survivors. Similarly, headache [n=220 (72.8 %)] followed by joint pain [n=58(19.2 %)] were the most prevalent among close contacts.

**Table 2:** Clinical profile of Ebola child survivors and contacts

Clinical Symptoms	Variables	EVD child survivor n (%) N=159	Close contacts n (%) N=303	Total n (%)	p-value
Chest Pain	Yes	47(29.6)	28(9.2)	75(16.2)	<0.001
	No	112(70.4)	275(90.8)	387(83.8)	
Joint Pain	Yes	101(63.5)	58(19.1)	159(34.4)	<0.001
	No	58(36.5)	245(80.9)	303(65.6)	
Muscle Pain	Yes	57(35.8)	39(12.9)	96(20.8)	<0.001
	No	103(64.2)	264(87.1)	366(79.2)	
Eye Pain	Yes	67(42.1)	20(6.6)	87(18.8)	<0.001
	No	92(57.9)	283(93.4)	375(81.2)	
Eye Redness	Yes	54(34.0)	20(6.6)	74(16.0)	<0.001
	No	105(66.0)	283(93.4)	388(84.0)	
Dry eyes	Yes	29(18.2)	11(3.6)	40(8.7)	<0.001
	No	130(81.8)	292(96.4)	422(91.3)	
Blurred Vision	Yes	22(13.8)	3(1.0)	25(5.4)	<0.001
	No	137(86.2)	300(99.0)	437(94.6)	
Sensitive to light	Yes	40(25.2)	12(4.0)	52(11.3)	<0.001
	No	119(74.8)	290(96.0)	410(88.7)	
Ringing in the ear (Tinnitus)	Yes	32(20.1)	11(3.6)	43(9.3)	<0.001
	No	127(79.9)	292(96.4)	419(90.7)	
Hearing Loss	Yes	33(20.8)	9(3.0)	42(9.1)	<0.001
	No	126(79.2)	294(97.0)	420(90.9)	
Epigastric Reflux	Yes	13(8.2)	2(0.7)	15(3.2)	<0.001
	No	146(91.8)	301(99.3)	447(96.8)	
Blood or mucus in the stool	Yes	15(9.4)	9(3.0)	24(5.2)	0.003
	No	144(90.6)	294(97.0)	438(94.8)	
Headache	Yes	138(86.8)	221(72.9)	359(77.7)	0.001
	No	21(13.2)	82(27.1)	103(22.3)	
Memory Impairment	Yes	29(18.2)	3(1.0)	32(6.9)	<0.001
	No	130(81.8)	300(99.0)	430(93.1)	
Loss of sensation	Yes	23(14.5)	6(2.0)	29(6.3)	<0.001
	No	136(85.5)	297(98.0)	433(93.7)	
Tremor	Yes	17(10.7)	4(1.3)	21(4.5)	<0.001
	No	142(89.3)	299(98.7)	441(95.5)	
Seizures	Yes	5(3.1)	5(1.7)	10(2.2)	0.302
	No	154(96.9)	298(98.3)	452(97.8)	
Mood Changes	Yes	72(45.3)	18(5.9)	90(19.5)	<0.001
	No	87(54.7)	285(94.1)	372(80.5)	

Table 3 provides results of the regression analysis of the self-reported symptoms between EVD child survivors and their close contacts. Joint pain (AOR=2.63; 95 % CI:1.31-5.28, p=0.006), hearing loss (AOR=3.85; 95 %CI: 1.15-12.87, p=0.029), memory impairment (AOR=7.76;0.95 %CI: 1.34-45.01 p=0.022), mood changes (AOR=5.07; 95 %CI: 2.35-10.94, p<0.001) were more common among EVD child survivors than their contacts. In addition, EVD child survivors were at least three times (AOR=6.77; 95 % CI: 3.51-13.06, p<0.001) more likely to have been hospitalised after the end of the Ebola outbreak than their contacts.

**Table 3:** Multivariate regression analysis of post–Ebola clinical symptoms

Self-reported symptoms	Variables	AOR	95 %CI	p-value
Joint pain	Yes	2.633	1.313-5.278	0.006
	No	1		
Muscle pain	Yes	0.452	0.193-1.059	0.068
	No	1		
Eye pain	Yes	4.559	2.156-9.639	<0.001
	No	1		
Hearing loss	Yes	3.849	1.151-12.871	0.029
	No	1		
Memory impairment	Yes	7.763	1.339-45.007	0.022
	No	1		
Loss of sensation	Yes	7.321	1.256-42.680	0.027
	No	1		
Seizures	Yes	0.120	0.013-1.112	0.062
	No	1		
Mood changes	Yes	5.070	2.350-10.939	<0.001
	No	1		
Hospitalised after the end of Ebola outbreak	Yes	6.774	3.513-13.062	<0.001
	No	1		
Hospitalised during the Ebola outbreak	Yes	3.257	0.862-12.303	0.082
	No	1		

AOR- Adjusted Odds Ratio

## Discussion

The study presents findings of the first-ever analysis of post-Ebola symptoms focusing purely on EVD child survivors. Our analysis indicates that EVD child survivors have higher odds of presenting with specific clinical symptoms and being hospitalised after the end of the Ebola outbreak than their close contacts. Musculoskeletal, ocular, auditory and neurological symptoms were more prevalent among Ebola survivors compared to their close contacts. Joint pain and headache were the most common self-reported symptoms among EVD child survivors. Our findings align with previous studies conducted among adult EVD survivors in West Africa [7, 9, 18–21]. We observed that EVD child survivors were more likely to present with joint pain, eye pain, and hearing loss compared to their contacts. Long term musculoskeletal, ophthalmic, and auditory sequelae have been demonstrated among adult EVD survivors, including children [7, 8, 14, 22, 23]. As in adult EVD survivors, these sequelae can limit their ability to be independent, perform daily life activities such as walking, running, going to school etc. Such limitations would have an adverse impact on their quality of life and wellbeing.

Our data has shown that EVD child survivors were more likely to be at higher odds of presenting with memory loss and loss of neurological sensation than their contacts. Such subjective post-EVD neurological impairment is a common sequela observed in previous studies among adult EVD child survivors [7, 9, 19, 24, 25]. Mood changes were also observed among this cohort of EVD child survivors. The mood changes could indicate a long-term neurological abnormality or common mental health disorders, such as severe depression, post-traumatic stress disorder and anxiety. Such symptoms of psychological distress have been reported among adult Ebola survivors [4, 26, 27].

Our multivariate analysis indicates that child survivors were more likely to have been hospitalised than their contacts after the end of the Ebola outbreak. This may be due to the weak health status of child survivors, which may be attributed to the high physical and psychological trauma they experienced during admission and after discharge from the Ebola treatment centres [4, 6]. Also, the increased usage of healthcare facilities following the end of the EVD outbreak may also be because EVD survivors were included as beneficiaries of the free healthcare initiative initially designed for pregnant women, lactating mothers, and children under five years [28, 29]. Our finding is in line with a recent study that reported EVD survivors’ increased utilisation of healthcare services after becoming recipients of the free healthcare initiative in Sierra Leone [10].

### Recommendations for practice

The following recommendations are suggested based on our findings discussed above. Routine screening, treatment and monitoring of these symptoms is required to prevent long-term disability among EVD child survivors. The interim guidance for clinical care of EVD survivors published by WHO could serve as a guide to managing these symptoms [3]. Also, EVD child survivors presenting with cognitive impairment and potential mental health disorders must have access to psychiatric assessment and, if possible, treatment to prevent long term neurological damage and mental health disorders. In addition, community, including school-based psychosocial support, is required to reduce the psychological trauma that EVD child survivors are experiencing.

### Study limitations

A key limitation of our study is the reliance on self-reports of the clinical manifestations among survivors and their contacts. Also, serological screening among contacts was not done to rule out asymptomatic EVD infection, although asymptomatic EVD infection is uncommon in West Africa [30]. Based on our inclusion criteria, we did not target all EVD childhood survivors in these two districts. In addition, we were unable to recruit EVD child survivors whom the Ministry of Social Welfare, Gender and Children’s Affairs (MSWGCA) and Sierra Leone Association of Ebola Survivors did not register. Furthermore, mood changes may influence self-reporting of physical health and symptomology, leading to the under-reporting of some of the physical symptoms. In our study, cases were older than controls, and as such would have made EVD survivors to better able to report symptoms such as memory impairment. The age difference may also explain the variation in the distribution of mood impairment rather than EVD.

Notwithstanding these limitations, our study presents the first-ever statistically significant case-controlled findings on post-Ebola symptoms solely among EVD child survivors. Further studies are needed to confirm our findings and to have a better insight into the underlying pathogenesis that explains the clinical manifestations observed in our study.

## Conclusion

Our study suggests that, like in adults, EVD child survivors compared to their close contacts have higher odds of experiencing musculoskeletal, ophthalmic, auditory and neurological symptoms more than a year after discharge from an Ebola treatment centre. Routine screening, treatment and monitoring of these symptoms, including child friendly psychosocial support, are required to prevent long-term disability among EVD child survivors. Further research is required to address our study limitations, to confirm our findings, and at the same time improve our understanding of the clinical pathology underlying the occurrence of the clinical complications among EVD child survivors.

## Supplementary Information

**Additional file 1.**

## Data Availability

All data will be available upon reasonable request from the corresponding author.

## Abbreviations

AOR: Adjusted Odd Ratio
EVD: Ebola Virus Disease
PHU: Peripheral Health Units
CHP: Community Health Post
MCHP: Maternal Child Health Post

## References

[1] WHO: Statement on the 1st meeting of the IHR Emergency Committee on the 2014 Ebola outbreak in West Africa. https://www.who.int/news/item/08-08-2014-statement-on-the-1st-meeting-of-the-ihr-emergency-committee-on-the-2014-ebola-outbreak-in-west-africa Accessed 26 March 2016. 2014.

[2] WHO: Ebola Situation Report - 30 March 2016. http://apps.who.int/ebola/current-situation/ebola-situation-report-30-march-2016. Accessed 16 October 2017 2016.

[3] WHO: Clinical care for survivors of Ebola virus disease: interim guidance. http://apps.who.int/iris/bitstream/10665/204235/1/WHO_EVD_OHE_PED_16.1_eng.pdf?ua=1. Accessed 4th September 2019. 2016.

[4] James PB, Wardle J, Steel A, Adams J. Post-Ebola psychosocial experiences and coping mechanisms among Ebola survivors: a systematic review. Trop Med Int Health. 2019;24(6):671–91. 10.1111/tmi.13226 30843627

[5] Lotsch F, Schnyder J, Goorhuis A, Grobusch MP (2017). Neuropsychological long-term sequelae of Ebola virus disease survivors - A systematic review. Travel medicine and infectious disease.

[6] Vetter P, Kaiser L, Schibler M, Ciglenecki I, Bausch DG (2016). Sequelae of Ebola virus disease: the emergency within the emergency. The Lancet Infectious diseases.

[7] Clark DV, Kibuuka H, Millard M, Wakabi S, Lukwago L, Taylor A, Eller MA, Eller LA, Michael NL, Honko AN (2015). Long-term sequelae after Ebola virus disease in Bundibugyo, Uganda: a retrospective cohort study. Lancet Infect Dis.

[8] PREVAIL III Study Group (2019). A Longitudinal Study of Ebola Sequelae in Liberia. New England Journal of Medicine.

[9] Bond NG, Grant DS, Himmelfarb ST, Engel EJ, Al-Hasan F, Gbakie M, Kamara F, Kanneh L, Mustapha I, Okoli A et al: Post-Ebola Syndrome Presents With Multiple Overlapping Symptom Clusters: Evidence From an Ongoing Cohort Study in Eastern Sierra Leone. Clin Infect Dis 2021. 10.1093/cid/ciab267 PMC8442780 33822010

[10] James PB, Wardle J, Steel A, Adams J (2020). Ebola survivors’ healthcare-seeking experiences and preferences of conventional, complementary and traditional medicine use: A qualitative exploratory study in Sierra Leone. Complementary Therapies in Clinical Practice.

[11] James PB, Wardle J, Steel A, Adams J, Bah AJ, Sevalie S: Providing healthcare to Ebola survivors: A qualitative exploratory investigation of healthcare providers’ views and experiences in Sierra Leone. Global Public Health 2020:1–16. 10.1080/17441692.2020.1762105 32379008

[12] Shah T, Greig J, van der Plas LM, Achar J, Caleo G, Squire JS, Turay AS, Joshy G, D’Este C, Banks E (2016). Inpatient signs and symptoms and factors associated with death in children aged 5 years and younger admitted to two Ebola management centres in Sierra Leone, 2014: a retrospective cohort study. The Lancet Global Health.

[13] Team WER (2015). Ebola virus disease among children in West Africa. New England J Med.

[14] Etard JF, Sow MS, Leroy S, Toure A, Taverne B, Keita AK, Msellati P, Magassouba N, Baize S, Raoul H (2017). Multidisciplinary assessment of post-Ebola sequelae in Guinea (Postebogui): an observational cohort study. The Lancet Infectious diseases.

[15] WHO-AFRO: WHO country cooperation strategy 2017-2021: Sierra Leone. https://apps.who.int/iris/handle/10665/258610 Accessed 23 July 2020. 2017.

[16] UNICEF: Country profiles- Sierra Leone: Maternal, neonatal and child health. https://www.unicef.org/sierraleone/maternal-neonatal-and-child-health. Accessed 16 June 2020. 2021.

[17] Ministry of Health and Sanitation-Sierra Leone: Annual Health Sector Performance Report 2016. https://www.afro.who.int/publications/sierra-leone-health-sector-performance-report-2016. Accessed 24 June 2018

[18] Mattia JG, Vandy MJ, Chang JC, Platt DE, Dierberg K, Bausch DG, Brooks T, Conteh S, Crozier I (2016). Fowler RAet al: Early clinical sequelae of Ebola virus disease in Sierra Leone: a cross-sectional study. Lancet Infect Dis.

[19] Qureshi AI, Chughtai M, Loua TO, Kolie JP, Camara HFS, Ishfaq MF, N’Dour CT, Beavogui K (2015). Study of Ebola Virus Disease Survivors in Guinea. Clin Infect Dis.

[20] Scott JT, Sesay FR, Massaquoi TA, Idriss BR, Sahr F, Semple MG (2016). Post-Ebola Syndrome, Sierra Leone. Emerg Infect Dis.

[21] Tiffany A, Vetter P, Mattia J, Dayer J-A, Bartsch M, Kasztura M, Sterk E, Tijerino AM, Kaiser L, Ciglenecki I (2016). Ebola Virus Disease Complications as Experienced by Survivors in Sierra Leone. Clin Infect Dis.

[22] Steptoe PJ, Scott JT, Harding SP, Beare NAV, Semple MG, Vandy MJ, Sahr F (2017). Ocular Complications in Survivors of the Ebola Outbreak in Guinea. Am J Ophthalmol.

[23] Rowe AK, Bertolli J, Khan AS, Mukunu R, Muyembe-Tamfum JJ, Bressler D, Williams AJ, Peters CJ, Rodriguez L, Feldmann Het al: Clinical, Virologic, and Immunologic Follow-Up of Convalescent Ebola Hemorrhagic Fever Patients and Their Household Contacts, Kikwit, Democratic Republic of the Congo. J Infect Dis 1999, 179(Supplement_1):S28-S35. 10.1086/514318 9988162

[24] Epstein L, Wong KK, Kallen AJ, Uyeki TM: Post-Ebola Signs and Symptoms in U.S. Survivors. New England J Med 2015, 373(25):2484-2486. 10.1056/NEJMc1506576 26672870

[25] Jagadesh S, Sevalie S, Fatoma R, Sesay F, Sahr F, Faragher B, Semple MG, Fletcher TE, Weigel R, Scott JT (2018). Disability Among Ebola Survivors and Their Close Contacts in Sierra Leone: A Retrospective Case-Controlled Cohort Study. Clin Infect Dis.

[26] Bah AJ, James PB, Bah N, Sesay AB, Sevalie S, Kanu JS (2020). Prevalence of anxiety, depression and post-traumatic stress disorder among Ebola survivors in northern Sierra Leone: a cross-sectional study. BMC Public Health.

[27] Secor A, Macauley R, Stan L, Kagone M, Sidikiba S, Sow S, Aronovich D, Litvin K, Davis N. Alva Set al: Mental health among Ebola survivors in Liberia, Sierra Leone and Guinea: results from a cross-sectional study. BMJ Open. 2020;10(5). 10.1136/bmjopen-2019-035217 PMC7259862 32461296

[28] Government of Sierra Leone: Sierra Leone Government directs that all health facilities should provide free health care for Ebola survivors. https://cocorioko.net/sierra-leone-government-directs-that-all-health-facilities-should-provide-free-health-care-for-ebola-survivors/. Accessed 27 November 2018. 2016

[29] Witter S, Brikci N, Harris T, Williams R, Keen S, Mujica A, Jones A, Murray-Zmijewski A, Bale B (2018). Leigh Bet al: The free healthcare initiative in Sierra Leone: Evaluating a health system reform 2010–2015. Int J Health Plan Manag.

[30] Glynn JR, Bower H, Johnson S, Houlihan CF, Montesano C, Scott JT, Semple MG, Bangura MS, Kamara AJ (2017). Kamara Oet al: Asymptomatic infection and unrecognised Ebola virus disease in Ebola-affected households in Sierra Leone: a cross-sectional study using a new non-invasive assay for antibodies to Ebola virus. Lancet Infect Dis.

