# Supplementary material for: Post-Ebola sequelae among Ebola child survivors in Sierra Leone
Source: BMC Pediatr. 2021 Oct 30;21:482. doi: 10.1186/s12887-021-02957-w (PMC8556876; doi:10.1186/s12887-021-02957-w)
Supplement: Supplementary file 1 — Additional file 1. [file 12887_2021_2957_MOESM1_ESM.docx]

| Question Number | Questions | Response Categories | |
| --- | --- | --- | --- |
| 1 | Case identification number |  | |
| 2 | Age(years) |  | |
| 3 | Sex | Male  Female | |
| 4 | Weight | Weight (Kg) -------------- | |
| 5 | Region/district | Western Area Urban (WAU)  Western Area Rural (WAR)  Kenema (KE) | |
| 6 | Social status | Orphan  Non-Orphan | |
| 7 | Education | None  Primary  Secondary | |
| 8 | Any history of chronic illness  (Known illness before Ebola) | Yes  No | |
| 9 | How many times has the child been Hospitalized before Ebola outbreak |  | |
| 10 | How many times hospitalized during the Ebola outbreak |  | |
| 11 | How many times Hospitalized after Ebola outbreak? |  | |
| 12 | Experienced these symptoms after Ebola outbreak?  *(Please tick where applicable)* | **Musculo-skeletal** | Chest pain  Joint pain    Muscle pain |
|  |  | **Ocular** | Eye pain  Eye Redness  Dry eyes  Blurry vision  Sensitive to light |
|  |  | **Auditory** | Ringing ear  Hearing loss |
|  |  | **Abdominal** | Epigastric pain  Reflux,  Blood or mucus in the stool, |
|  |  | **Neurological** | Headache  Memory impairment  Loss of sensation  Tremor  Seizures |
|  |  | **Mental health** | Mood Changes |

Name of Data Collector__________________________________

SIGN ________________
